# Transcriptomic analyses reveal molecular mechanisms underlying growth heterosis and weakness of rubber tree seedlings

**DOI:** 10.1186/s12870-017-1203-3

**Published:** 2018-01-09

**Authors:** Hong Yang, Xuncheng Wang, Yongxuan Wei, Zhi Deng, Hui Liu, Jiangshu Chen, Longjun Dai, Zhihui Xia, Guangming He, Dejun Li

**Affiliations:** 1 Key Laboratory of Biology and Genetic Resources of Rubber Tree, Ministry of Agriculture, Rubber Research Institute, Chinese Academy of Tropical Agricultural Sciences, Baodao Xincun, Danzhou, Hainan 571737 China; 2 State Key Laboratory of Protein and Plant Gene Research, Peking-Tsinghua Center for Life Sciences, School of Advanced Agriculture Sciences and School of Life Sciences, Peking University, No. 5 Yiheyuan Road, Haidian District, Beijing, 100871 China; 3 Hainan Key Laboratory for Sustainable Utilization of Tropical Bioresources, College of Agriculture, Hainan University, Haikou, Hainan 570228 China

**Keywords:** *Hevea brasiliensis*, Transcriptome analyses, Growth heterosis, Molecular mechanism, Gene action model

## Abstract

**Background:**

Breeding rubber tree seedling with growth heterosis is vital for natural rubber production. It is the prerequisites for effectively utilizing growth heterosis to elucidate its molecular mechanisms, but the molecular mechanisms remain poorly understood in rubber tree. To elucidate seedling growth heterosis, we conducted comparative transcriptomic analyses between the two hybrids and their parents.

**Results:**

By identifying and comparing differently expressed genes (DEGs), we found that the hybrids (BT 3410 and WC 11) show significantly differential expression profiles from their parents (PR 107 and RRIM 600). In BT 3410-parent triad, 1092 (49.95%) and 1094 (50.05%) DEGs indicated clear underdominance or overdominance, respectively. Whereas in WC 11-parent triad, most DEGs (78.2%, 721) showed low- or high-parent dominance; 160 (17.35%) exhibited expression patterns that are not statistically distinguishable from additivity, and 8 (0.87%) and 33 (3.58%) DEGs exhibited underdominance and overdominance, respectively. Furthermore, some biological processes are differentially regulated between two hybrids. Interestingly, the pathway in response to stimulus is significantly downregulated and metabolic pathways are more highly regulated in BT 3410.

**Conclusions:**

Taken together, the genotypes, transcriptomes and biological pathways (especially, carbohydrate metabolism) are highly divergent between two hybrids, which may be associated with growth heterosis and weakness. Analyzing gene action models in hybrid-parent triads, we propose that overdominance may play important roles on growth heterosis, whereas dominance on hybrid weakness in rubber tree seedlings. These findings bring new insights into our understanding of growth heterosis of rubber tree seedling.

**Electronic supplementary material:**

The online version of this article (doi: 10.1186/s12870-017-1203-3) contains supplementary material, which is available to authorized users.

## Background

Heterosis, or hybrid vigor, refers to the phenomenon that hybrid progeny indicates greater biomass, yield, speed of development, or other agronomic traits than one or both parents [1]. Since heterosis was first described by Charles Darwin [2], it has been widely exploited in plant breeding to dramatically improve the production of crop plants [3]. In contrast to heterosis utilization, the molecular mechanisms involved in this basic biological phenomenon are not well understood.

Three main genetic models are proposed to explain heterosis [1, 4]. The dominance model states that a complementing action of slightly deleterious recessive alleles contributes to heterosis in F_1_ hybrid [5]. In contrast to dominance model, overdominance proposes that heterosis comes from favorable allelic interactions at heterozygous loci [6]. Epistasis coming from gene-by-gene interactions is a third genetic model to explain heterosis [7]. In corn and rice, epistasis is associated with inbreeding depression and heterosis [8–10]. Except for some examples from heterozygote advantage and single-locus heterosis, one of three models can not by itself adequately explain heterotic phenomena. Some rice hybrids indicate different genetic models, including epistasis [7], dominance [11], overdominance [12], and overdominance as well as pseudo-dominance [13]. Moreover, similar results have been reported in wheat [14], maize [15–17], upland cotton [18], and *Arabidopsis* [19, 20]. Differential gene expression in parents and hybrids may be responsible for heterosis [21–28]. In contrast to its parents, a hybrid usually indicates expression levels of genes equal to the mid-parent (additivity, AD), the high or low parent (high or low parent dominance, HPD or LPD), above the high parent (overdominance, OD), or below the low parent (underdominance, UD).

As a critical raw material, natural rubber (NR) can be utilized to manufacture >40,000 products. However, it cannot be replaced by synthetic alternatives due to its unique properties including resilience, elasticity, impact and abrasion resistance, efficient heat dispersion and malleability at cold temperature [29]. Among >2000 varieties of rubber-producing plants, the Brazilian rubber tree (*Hevea brasiliensis*) is the only species cultivated commercially for harvesting NR. The demand for NR has continuously increased due to fast development of the world economy. Breeding high-yield clones is an effective means to meet world demand on NR. Rubber tree breeding has resulted in a gradual increase in NR yields from 650 kg/ha in unselected seedlings to 1600 kg/ha in optimised hybrid varieties, however the yield of the most productive varieties is still much inferior to the theoretical yield, which was predicted to be 7000–12,000 kg/ha [30].

Besides high-yield rubber tree varieties, rubber tree seedling with growth heterosis is vital for NR production. Compared with rubber tree seedlings with growth weakness, those with growth heterosis facilitate themselves to survive various biotic and abiotic stresses; therefore rubber tree seedlings with growth heterosis indicate higher survival ratio than those with growth weakness. Rubber tree usually experiences an initial growth phase varying generally from 5 to 7 years. Trees are tapped when their trunks attain 50 cm in girth, therefore rubber tree seedlings with growth heterosis can shorten the initial unproductive period. In addition, rubber tree seedling with growth heterosis can shorten the period of seedling cultivation, which can improve the efficiency of breeding seedlings. Some researchers reported that the seedlings of different rubber tree varieties indicated significant difference of growth traits in their immature periods [31–33]. In addition, Sankariammal and Mydin reported that the Wickham × Amazonian hybrids indicated a significant yield increase (14–82%) [34]. Although it is vital for NR production to breed rubber tree seedlings with growth heterosis, molecular mechanisms underlying growth heterosis and weakness of rubber tree seedlings are not reported until now.

Here, we generated transcriptome profiles of two rubber tree varieties, and their two F_1_ hybrids which show growth heterosis and weakness, respectively. The gene action models in two hybrid-parent triads were systematically compared to illustrate their relationship with growth heterosis and weakness. We found that underdominance and overdominance expression patterns, and differential expression of genes involved in the pathways of in response to stimulus, and carbohydrate metabolism may contribute to growth heterosis of rubber tree seedlings.

## Methods

### Plant materials, seedlings girth and height, and extraction of RNA

Five rubber tree varieties, PR 107, RRIM 600, BT 3410, RY 7–33-97, and WC 11 were planted in National Rubber Tree Germplasm Repository, Hainan, China. The leaves from the five varieties were collected with three biological replicates. Two and three replicates were used for RNA-seq and qRT-PCR, respectively. RNA was isolated according to the methods described by Doyle [35]. RNA qualities were assayed with a 2100 Bioanalyzer (Agilent Technologies). In addition, the girth and height of one-year-old seedlings were measured at the height of 1.0 m and seedling top above the ground level on December 28, 2014, respectively. The seedlings girth and height are shown as mean ± S.D. Phenotype difference among tree varieties were calculated using *p* < 0.05 in One-Way ANOVA after Tukey HSD Post-hoc test correction in SPSS (SPSS19.0, IBM, Armonk, New York).

### Illumina sequencing, assembly, and annotation

Sequencing library construction and Illumina sequencing were performed at Novogene Corporation, China. Briefly, mRNA with poly (A) tail was isolated and purified from total RNA with Sera-mag magnetic oligo (dT) beads (Illumina). After the purified mRNA was fragmented into small pieces with 100–400 bp length, the double-stranded cDNA was synthesized using the SuperScript double-stranded cDNA synthesis kit with random hexamer primers (Illumina). The synthesized cDNA was performed with end-repair and phosphorylation, and then the cDNA fragments were 3′ adenylated using Klenow Exo- (3′ to 5′ exo minus, Illumina). Next, the ends of the aforementioned 3′-adenylated cDNA fragments were linked with Illumina paired-end adapters. The linked products mentioned above were purified by 2% agarose gel, and then the cDNA fragments with ~200 bp were excised from the gel. The purified cDNA templates were enriched by PCR amplification using PCR primer PE 1.0 and 2.0 (Illumina) with phusion DNA polymerase. In the end, the cDNA library with 200 bp insertion fragment was obtained and sequenced using Illumina HiSeqTM 2000. The sequencing workflow was as following: template hybridization, isothermal amplification, linearization, blocking, sequencing primer hybridization, and sequencing on the sequencer for read 1. After finishing the first read, the templates can be regenerated in situ to perform a second read from the opposite end of the fragments. Once the original templates are cleaved and removed, the reverse strands undergo sequencing-by-synthesis. Raw data (raw reads) of fastq format were firstly processed through in-house perl scripts. We filtered out low-quality reads according to three conditions: (1) reads with the adaptor sequence; (2) the percentage of low-quality bases (Qphred ≤20) was >50% in a read; (3) the percentage of N bases was ≥10% in a read, and then the paired-end reads were assembled according to the Trinity (V 2.0.6) with min_kmer_cov set to 2 by default and all other parameters set default [36]. The assembled unigenes were functionally annotated based on the following databases: Nr (NCBI non-redundant protein sequences), Nt (NCBI non-redundant nucleotide sequences), Pfam (Protein family), KOG/COG (Clusters of Orthologous Groups of proteins), Swiss-Prot (A manually annotated and reviewed protein sequence database), KO (KEGG Ortholog database), and GO (Gene Ontology).

### qRT-PCR analysis

The gene-specific primers of 23 unigenes and internal reference gene were listed in Additional file 1: Table S1. The qRT-PCR conditions were as follows: 94 °C for 30 s for denaturation, followed by 45 cycles at 94 °C for 5 s, 60 °C for 20 s, and 72 °C for 20 s. The relative abundance of each unigene was calculated with LightCycler Relative Quantification Software 4.05. All qRT-PCR experiments were repeated 3 times using independent cDNA preparations, and the values are presented as mean ± S.D. The statistical analysis was performed using SPSS software version 19.0 to find significant difference (*p* < 0.05) with One-Way ANOVA and Tukey HSD Post-hoc test correction (IBM, Armonk, New York).

### Identification and annotation of DEGs, as well as models of gene action

The expression level of each gene was calculated using RSEM software (v1.1.17) [37]. With the assembled unigenes as reference, the clean raw reads from four rubber trees varieties were separately realigned to the assembled transcriptome. DESeq R package (1.10.1) was utilized to identify the DEGs with a padj ≤0.05 [38]. If gene expression is different between hybrids and one of their parents, the gene is defined as DEG. The enrichment analysis of GO functional terms and KEGG pathways of the DEGs were calculated as the ratio of the relative occurrence in a set of genes to the relative occurrence in the genome. The statistical significance of the functional enrichment within gene sets was evaluated using the hypergeometric distribution adjusted by the Bonferroni correction for multiple hypotheses testing. Adjusted *P* values <0.05 were considered as significant. In addition, gene expression levels were log_2_ transformed and used to generate heatmap using R (http://www.r-project.org).

According to the method of Rapp et al. [39], the DEGs among a hybrid-parent triad were classified into 5 possible expression classes of differential expression, that is, expression levels of genes equal to the mid-parent (additivity), the high or low parent (high or low parent dominance), above the high parent (overdominance), or below the low parent (underdominance). In addition, the expression profiles of 23 randomly selected genes were also classified into the aforementioned gene action models in a hybrid-parent triad.

## Results

### Transcriptome profiling of two rubber tree varieties and their hybrids

**Fig. 1:**
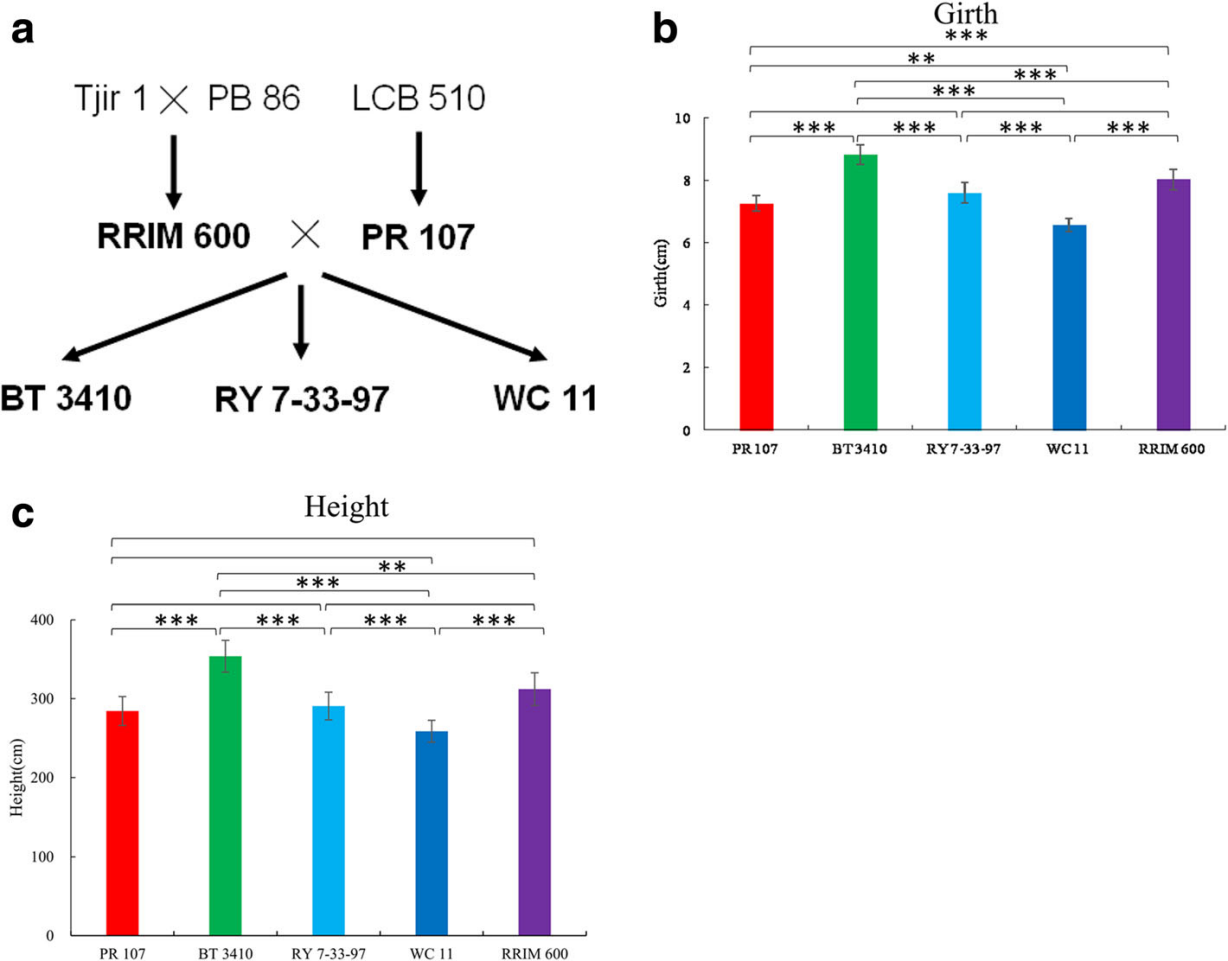
Genetic background, seedling girth and height of five rubber tree varieties. **a** Genetic background of rubber tree varieties analyzed in this study. Female parent of each cross is listed first. The five varieties analyzed in this study are in bold type. **b**, **c** Seedling girth and height of three hybrids and their parents. The data is firstly analyzed with One-Way ANOVA, and then analyzed with Tukey HSD Post-hoc test. Three and two asterisks represent *p* < 0.0001 and *p* < 0.001, respectively

To obtain insight into changes in gene expression and regulatory networks that can affect on growth differences among rubber trees, we generated transcriptome profiles of two rubber tree varieties PR 107, RRIM 600, and their F_1_ hybrids BT 3410 and WC 11 using Illumina RNA-seq. PR 107 (a primary variety), and RRIM 600 (a secondary variety), are cultivated from the unselected seedling of clone LCB 510 and the offsprings of two primary clones (Tjir 1 and PB 86), respectively. BT 3410, RY 7–33-97 and WC 11 are generated by selecting from the F_1_ offsprings of PR 107 and RRIM 600 (Fig. 1a). The seedling girth of RRIM 600 is significantly thicker than that of PR 107 (Fig. 1b and Additional file 2: Table S2). The seedling height of RRIM 600 is taller than that of PR 107, but the difference is not statistically significant (Fig. 1c and Additional file 2: Table S2). Compared to their parents, BT 3410 is significantly thicker and taller than RRIM 600, whereas WC 11 is shorter and thinner than PR 107 (Fig. 1b, c and Additional file 2: Table S2), indicating growth heterosis and weakness in BT 3410 and WC 11, respectively. Different from BT 3410 and WC 11, the seedling girth and height of RY 7–33-97 are between PR 107 and RRIM 600 (Fig. 1b and c, Supplementary Dataset 2).

**Fig. 2:**
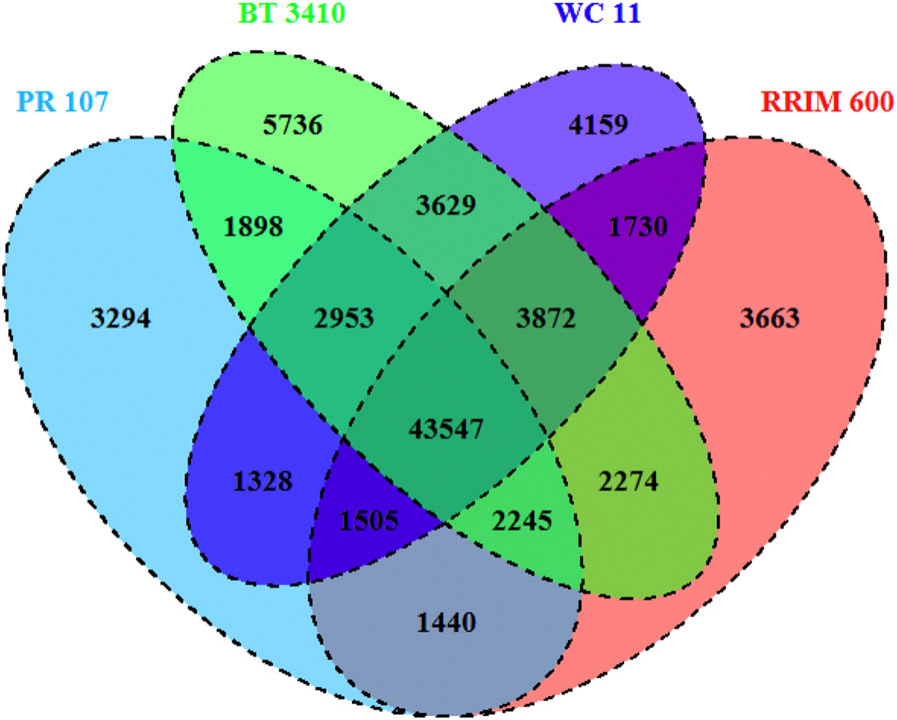
Comparison of expression gene numbers among four rubber tree varieties using Venn diagram

Leaves from one-year-old seedling were used to generate RNA-seq libraries. After the adaptors, low-quality and contaminated reads were removed, a total of 331,788,346 RNA-seq reads were obtained from PR 107, RRIM 600, BT 3410, and WC 11. We obtained 152,231 unigenes by using high-quality reads from all four rubber tree varieties for de novo transcriptome assembling. The average and N50 lengths of the assembled unigenes are 731 bp and 1193 bp, respectively. Of 152,231 unigenes, 13,277 (~8.72%) and 96,971 (~63.69%) indicate homology to the genes or their protein products from all or at least one of seven databases including NCBI Nr and Nt, Pfam, KOG/COG, Swiss-prot, KEGG, and GO with e-value cutoff of 1e-5, respectively (Table 1). With the 152,231 assembled unigenes as reference, the high-quality clean reads from the four varieties were separately realigned, and gene expression was calculated based on mapped reads in each variety. We found that there are 58,210, 60,276, 66,514, and 62,723 genes that were expressed in PR 107, RRIM 600, BT 3410, and WC 11, respectively, and 43,547 genes were expressed in all four rubber tree varieties (Fig. 2).

**Table 1:** Annotated percentage of unigenes in different databases

Items	Number of Unigenes	Percentage (%)
Annotated in NR	82,098	53.92
Annotated in NT	75,970	49.9
Annotated in KO	28,265	18.56
Annotated in SwissProt	57,008	37.44
Annotated in PFAM	49,901	32.77
Annotated in GO	52,096	34.22
Annotated in KOG	28,773	18.9
Annotated in all Databases	13,277	8.72
Annotated in at least one Database	96,971	63.69
Total Unigenes	152,231	100

### BT 3410 and WC 11 show significantly different models of gene action

**Fig. 3:**
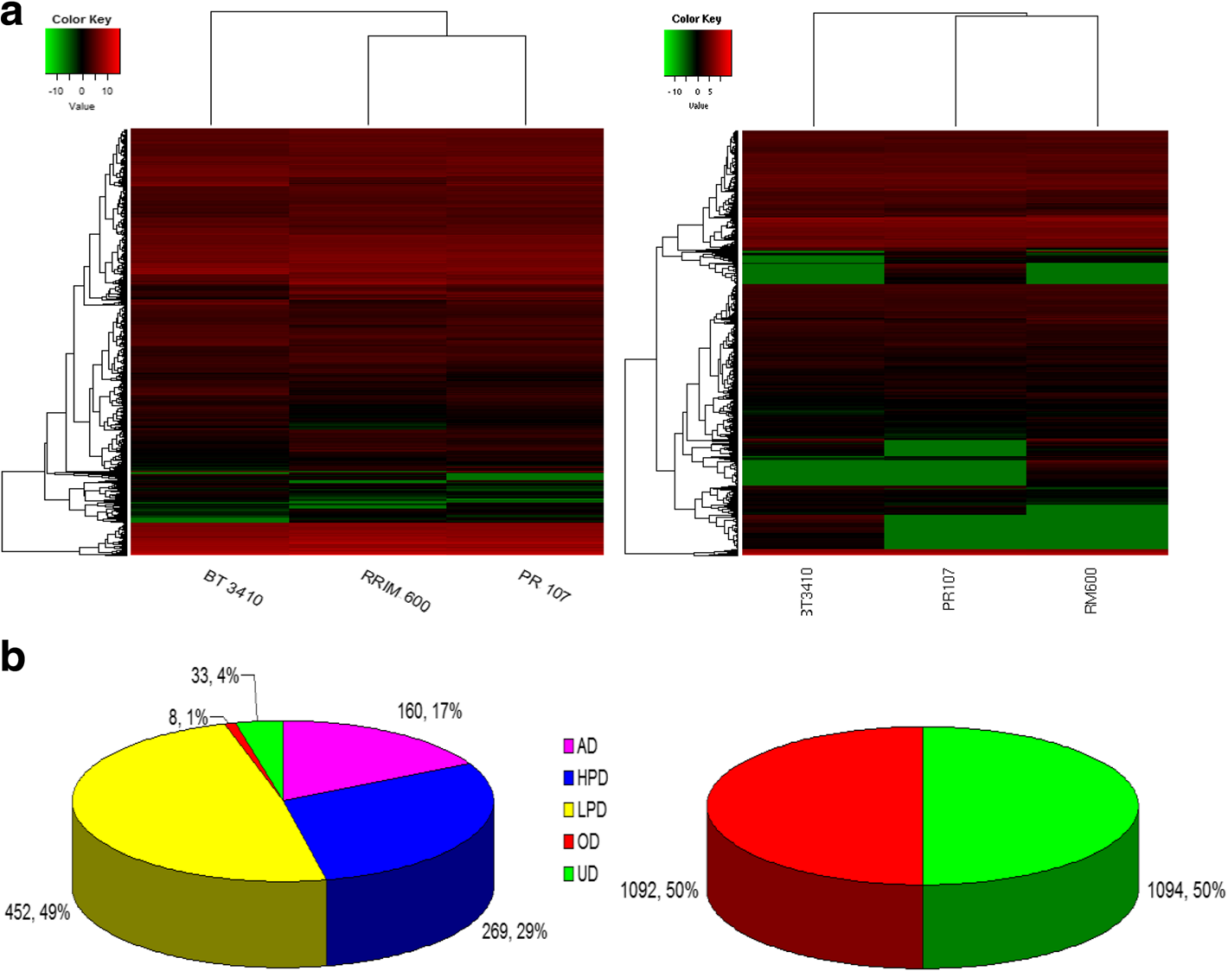
Heat map and gene action models in two hybrid-parent triads. **a** Heat map generated from gene expression in two hybrid-parent triads. **b** Sector graph of gene action models in WC 11 (left) and BT 3410 (right)

Next, we investigated the variation in gene expression among hybrids and parents. With a padj <0.05 as a threshold, approximately 1.95% (2975) unigenes were identified as the differently expressed genes (DEGs) between RRIM 600 and PR 107, and about 6.17% (9396) and 6.37% (9696) unigenes were identified as the DEGs among a BT 3410- or WC 11-parent triad, respectively. We investigated the expression profiles of DEGs in two hybrid-parent triads using hierarchical clustering. As shown in Fig. 3a, gene expression profiles between PR 107 and RRIM 600 differ from each other. BT 3410 and WC 11 show significantly differential expression profile from both parents, which, to some extent, may explain growth heterosis and weakness in BT 3410 and WC 11, respectively.

**Fig. 4:**
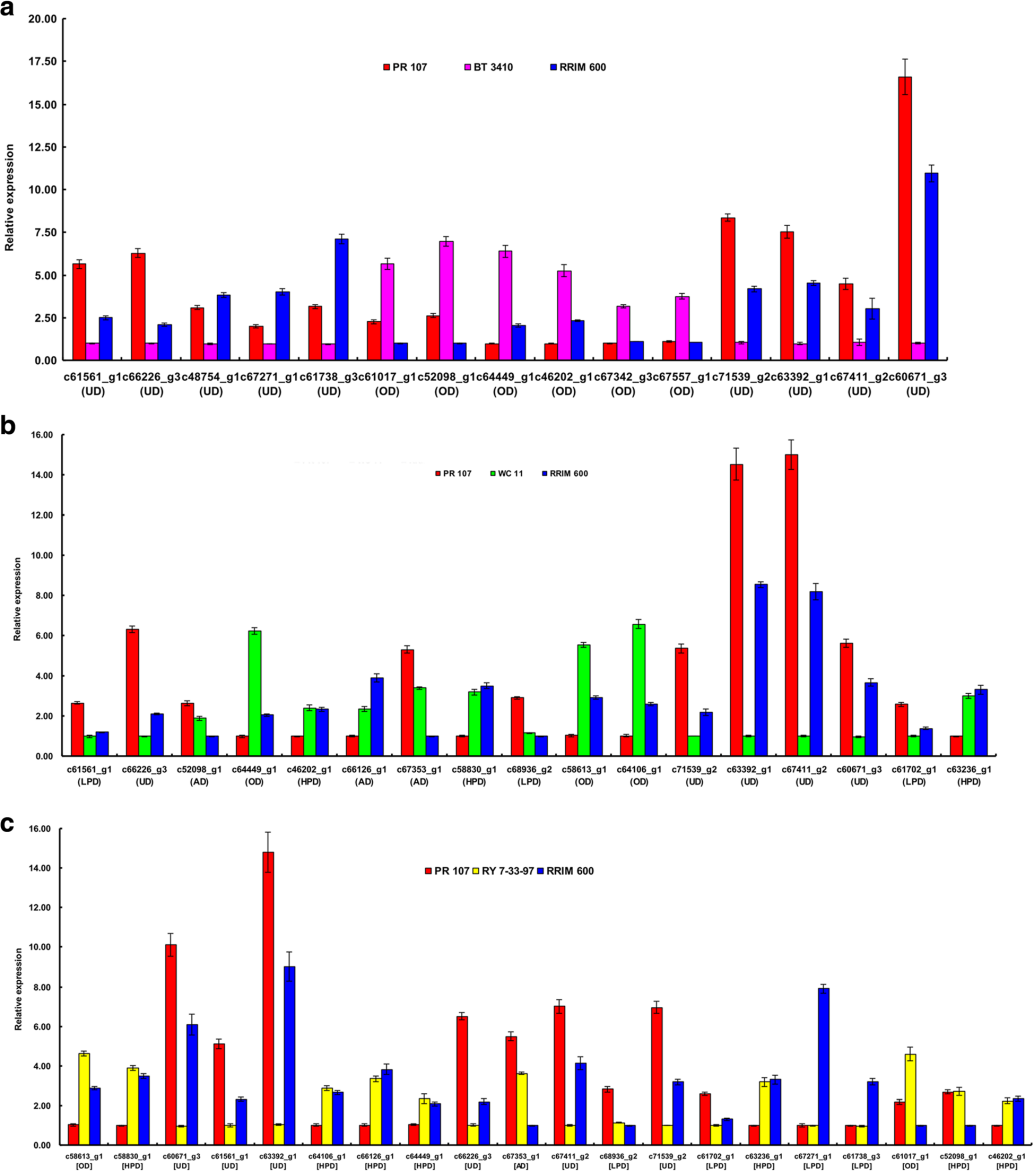
Gene expression profiles of 23 genes generated from qRT-PCR in three hybrid-parent triads. **a**, **b**, and **c** present gene expression profiles in BT 3410-, WC 11-, and RY 7-33-97-parent triads, respectively. AD, OD, UD, LPD, and HPD in parentheses and bracket refer to gene action models generated from RNA-seq and qRT-PCR analyses, respectively. One-Way ANOVA followed with Tukey HSD Post-hoc test and *p* < 0.05 were used to calculating gene expression difference

We further investigated the models of gene action in two hybrid-parent triad. In BT 3410, 2186 genes exhibit nonadditive model and no gene exhibits additive model. Of 2186 genes, 1092 (49.95%) and 1094 (50.05%) show UD and OD patterns, respectively (Fig. 3b and Additional file 3: Table S3). In WC 11, 992 genes could be classified into five gene model, among which only 8 (0.87%) and 33 (3.58%) exhibited UD and OD patterns, respectively. A large proportion of the genes (78.2%, 721) showed dominance models. Of these 721 genes, 269 and 452 exhibited HPD and LPD patterns, respectively. In addition, 160 genes (17.35%) exhibited expression patterns that are not statistically distinguishable from AD (Fig. 3b and Additional file 4: Table S4). We randomly selected 23 genes to validate their gene action models among two hybrid-parent triads using qRT-PCR. Among 23 genes (32 gene action models) deduced from RNA-seq data, all were confirmed by qRT-PCR (Fig. 4), suggesting that the gene action models analyzed in two hybrid-parent triads were highly reliable in our study. In addition, gene action models of the 23 genes were also analyzed in RY 7–33-97 and its parents by qRT-PCR. Among 20 genes indicating gene action models, 7, 4, 2, 1, and 6 genes indicated HPD, LPD, OD, AD, and UD gene action models in RY 7–33-97 and its parent triad, respectively (Fig. 4c). These results indicated that BT 3410, RY 7–33-97 and WC 11 show significantly different gene action models although they are the offsprings of the same parents, which may contribute to growth heterosis and weakness of seedlings in these rubber tree hybrids, respectively.

### Biological processes are differentially regulated between BT 3410 and WC 11

To explore the biological implication of changed gene expression in the two hybrids, we further investigated the Gene Ontology (GO) functional categories of genes with different expression patterns. In BT 3410, four GO terms including single-organism process, metabolic process, localization, and cellular process are significantly enriched for genes both with UD and OD patterns; GO term response to stimulus is significantly enriched specifically for genes with UD pattern, whereas seven other GO terms (biological regulation, cellular component organization or biogenesis, regulation of biological process, locomotion, positive regulation of biological process, biological adhesion, and multi-organism process) are enriched specifically for genes with OD pattern (Table 2). In WC 11, two GO terms (multi-organism process, and reproductive process) are significantly enriched for genes both with AD and LPD; two GO terms (multicellular organismal process, and response to stimulus), two GO terms (single-organism process, and immune system process), and two other GO terms (metabolic process, and positive regulation of biological process) are significantly enriched specifically for genes with AD, UD, and LPD patterns, respectively (Table 2). In addition, no GO term is significantly enriched for genes with HPD. Table 2 also shows that there are 5 GO terms (single-organism process, metabolic process, response to stimulus, multi-organism process and positive regulation of biological process) shared by the two hybrids. Interestingly, the genes involved in response to stimulus are significantly down-regulated (with UD pattern) in BT 3410, which potentially contribute to the seedling heterosis in this hybrid through tradeoff between stress response and growth. By contrast, in WC 11 exhibiting hybrid weakness, the genes involved in response to stimulus are equal to mid-parent values (with AD pattern). Furthermore, genes involved in positive regulation of biological process are significantly up-regulated (with OD pattern) in BT 3410, whereas are significantly down-regulated (with LPD pattern) in WC 11. Genes involved in metabolic process are up- or down-regulated in BT 3410, whereas in WC 11 only down-regulation is observed for genes in this process. Difference in the regulation of these pathways may also contribute to the growth difference between two rubber tree hybrids.

**Table 2:** Enriched GO terms within biological process in BT 3410 and WC 11

GO accession	Terms	Genes No. in this term	Genes No. in all terms	pvalue	qvalue	Models	Varieties
GO:0044699	single-organism process	463	825	1.11E-16	3.16E-15	UD	BT 3410
GO:0008152	metabolic process	555	825	2.55E-10	4.85E-09		
GO:0050896	response to stimulus	130	825	0.00027	0.00193		
GO:0051179	localization	156	825	0.00173	0.00987		
GO:0009987	cellular process	494	825	0.00736	0.03812		
GO:0044699	single-organism process	418	824	2.02E-07	5.75E-06	OD	
GO:0008152	metabolic process	535	824	7.76E-07	1.47E-05		
GO:0009987	cellular process	515	824	3.41E-05	0.00024		
GO:0065007	biological regulation	187	824	3.33E-05	0.00024		
GO:0071840	cellular component organization or biogenesis	102	824	0.00017	0.00095		
GO:0051179	localization	157	824	0.00122	0.00366		
GO:0050789	regulation of biological process	164	824	0.00158	0.00451		
GO:0040011	locomotion	13	824	0.00402	0.01091		
GO:0048518	positive regulation of biological process	16	824	0.00588	0.01525		
GO:0022610	biological adhesion	11	824	0.0113	0.028		
GO:0051704	multi-organism process	47	824	0.01814	0.03998		
GO:0051704	multi-organism process	16	106	2.41E-06	0.00014	AD	WC 11
GO:0032501	multicellular organismal process	8	106	7.85E-05	0.00224		
GO:0022414	reproductive process	4	106	0.00049	0.00929		
GO:0050896	response to stimulus	21	106	0.00579	0.04127		
GO:0051704	multi-organism process	27	325	0.00036	0.00508	LPD	
GO:0022414	reproductive process	7	325	0.00091	0.00861		
GO:0008152	metabolic process	212	325	0.00082	0.00861		
GO:0048518	positive regulation of biological process	8	325	0.00706	0.03657		
GO:0044699	single-organism process	16	23	0.00201	0.03818	UD	
GO:0002376	immune system process	1	23	0.00648	0.04106		

### Metabolic pathways are highly regulated in BT 3410

We also classified DEGs with different models of gene action in hybrids according to their KEGG pathways. As shown in Table 3, the DEGs in BT 3410 and in WC 11 are assigned to 31 and 27 KEGG pathways, respectively. With qvalue <0.05 as a threshold, 8 and 2 KEGG pathways are significantly enriched in BT 3410 and WC 11, respectively. Interestingly, all 10 enriched KEGG pathways in two hybrids belong to metabolism. Two KEGG pathways, amino acid metabolism and biosynthesis of other secondary metabolites, are enriched both in BT 3410 and WC 11. Six KEGG pathways including carbohydrate metabolism, lipid metabolism, metabolism of cofactors and vitamins, xenobiotics biodegradation and metabolism, metabolism of other amino acids, and overview are enriched specifically in BT 3410. It should be noted that carbohydrate metabolism is enriched in BT 3410, but not in WC 11. Carbohydrate metabolism is very important for generating cellular energy and various metabolites that provide material and energy to support plant growth and development. Therefore, the DEGs in carbohydrate metabolism pathway may directly contribute to the growth heterosis in rubber tree.

**Table 3:** The KEGG functional categories of the DEGs between two hybrid-parent triads

Functional categories	The gene number	
	BT 3410	WC 11
Metabolism		
Biosynthesis of other secondary metabolites	42^a^	14^a^
Amino acid metabolism	69^a^	20^a^
Carbohydrate metabolism	90^a^	13
Overview	67^a^	6
Metabolism of cofactors and vitamins	33^a^	4
Lipid metabolism	44^a^	13
Metabolism of other amino acids	29^a^	6
Xenobiotics biodegradation and metabolism	13^a^	5
Metabolism of terpenoids and polyketides	18	7
Energy metabolism	47	9
Nucleotide metabolism	15	3
Glycan biosynthesis and metabolism	3	1
Cellular Processes		
Cell growth and death	27	6
Transport and catabolism	32	2
Cell motility	5	0
Cellular commiunity	9	1
Environmental Information Processing		
Membrane transport	3	1
Signal transduction	39	15
Genetic Information Processing		
Replication and repair	11	5
Folding, sorting and degradation	23	9
Transcription	2	4
Translation	17	2
Organismal Systems		
Digestive system	10	2
Endocrine system	25	4
Development	4	0
Sensory system	1	0
Circulatory system	2	2
Environmental adaptation	11	6
Excretory system	2	0
Immune system	10	5
Nervous system	12	5

^a^denote function categories of the DEGs significantly enriched with q value <0.05

## Discussion

RNA sequencing (RNA-Seq) is a valuable tool for profiling expressed genes in plants and other organisms [40–42]. Leaf is an important tissue where photosynthesis and transpiration occur in rubber tree. The product of photosynthesis can provide material basis for rubber tree growth and development. Compared with rubber tree latexes [43–50] and barks [51–54], the research on transcriptome sequencing and assembly of rubber tree leaves was very limited. A total of 98,796 unigenes were obtained via the de novo assembly of RNA-seq data from rubber tree leaves [55]. The number of unigenes generated in this study was more than that reported by Fang et al. [55]. The N50 length of the unigenes in the present study was 1139 bp, which was longer than that obtained by Fang et al. [55]. Our leaf transcriptome data was submitted to NCBI database, which will further enrich the rubber tree transcriptome data. In addition, it will be helpful for developing molecular markers, improving transcriptome and genome assembly, gene cloning, etc.

Although three F_1_ progenies are generated form the same parents (RRIM 600 and PR 107), the F_1_ progenies, BT 3410, WC 11, and RY 7–33-97, show growth heterosis, growth weakness, and in between (represented by girth and height) relative to their parents, respectively. The growth differences among three F_1_ progenies may attribute to their parents’ heterozygosity. We found that a large proportion of genes (>82%) indicate nonadditive models in the two hybrids from heterozygous crossing, which is consistent with Hedgecock et al. [56], Wei et al. [23], Song et al. [57], and Li et al. [25], but differ from Swanson-Wagner et al. [15] and Stupar and Springer [58] who found very little gene expressions that were nonadditive or that exceeded parental levels in hybrids from homozygous crossing. On the other hand, the two hybrids also show different models of gene action. All the genes exhibit OD or UD actions in BT 3410 generated from heterozygous crossing, suggesting that OD or UD may play critical important roles in growth heterosis. As for the hybrids generated from homozygous crossing, Hedgecock et al. [56] proposed that the hybrid family with the greater degree of growth heterosis showed a greater OD proportion. Li et al. also reported that higher proportions of nonadditive and expression higher or lower than either parent were found in heterotic hybrids compared to a non-heterotic hybrid [59]. Different from BT 3410, additive and nonadditive models are separately ~17.35% and 82.65% in WC 11. Of the genes exhibiting nonadditive, ~78.2% exhibit LPD or HPD. Moreover, UD and OD are also observed in WC 11. Our results are broadly similar to those of Song et al. [57] and Li et al. [25], but their results are form the hybrids of homozygous crossing and the hybrids indicate heterosis. However, the hybrid WC 11 shows growth weakness, suggesting that a large proportion of dominance models may be associated with growth weakness of rubber tree seedlings. Additionally, the gene action models of the 23 genes randomly selected in RY 7–33-97 were different from those of WC 11 and BT 3401. Although the 23 gene action models could not necessarily represent the gene action models of RY 7–33-97 on transcriptome level, the seedling girth and height of RY 7–33-97 are indeed between PR 107 and RRIM 600. These results suggest that heterosis from homozygous and heterozygous crossings may possess some similarities and differences in models of gene action, which need further be validated in future experiments.

Different combinations among altered activity of biological processes can be charge of heterotic phenotypes [3, 60], which was further validated by Groszmanna et al. in *Arabidopsis* F_1_ hybrids. They suggested that the altered expression patterns of defense and stress response genes contributed to the greater growth of the hybrids [61]. In intraspecific hybrids, genome-wide expression of biotic and abiotic stress-responsive genes is diurnally repressed, which correlates with biomass heterosis and biomass quantitative trait loci [62]. It is worth noting that the DEGs significantly enriched in response to stimulus are separately classified in UD and AD in BT 3410 and WC 11. Given the antagonistic relationship between plant growth and defense responses [63–65], we suggest that these downregulated expressions of genes involved in response to stimulus may contribute to seedlings growth heterosis of the hybrids. Consistent with the aforementioned speculation, the genes involved in response to stimulus indicate AD model in WC 11 showing seedlings growth weakness. Besides response to stimulus terms, Wang et al. also found that the DEGs involved in metabolic process contribute to growth heterosis in both the hybrid and the hybrid mimic lines [66]. In accordance with Wang et al. [66], we found that the DEGs were also significantly enriched in metabolic process in BT 3410 (UD or OD) and WC 11 (LPD).

Lisec et al. reported that primary metabolism is a network that is closely linked to plant growth and development [67]. In a parallel study, they found that a linear combination of metabolite levels was shown to significantly correlate with biomass heterosis [68]. Consistent with the aforementioned results, we found that the DEGs in two hybrids significantly enriched in metabolism that is exclusive among five main KEGG pathways. We found that 87.5% and 100% enriched-KEGG pathways are primary or secondary metabolism in hybrids BT 3410 and WC 11, respectively. Yield heterosis-associated genes significantly enriched in carbohydrate metabolism in maize [17]. In addition, carbohydrate metabolism was identified as significant difference between LYP9 and its parents [23, 57, 69, 70]. In our study, carbohydrate metabolism significantly enriched in BT 3410 (growth heterosis), but not in WC 11 (growth weakness), suggesting that carbohydrate metabolism may contribute to growth heterosis. Given the aforementioned results, we suggest that the DEGs involved in the enriched carbohydrate metabolism may work together to be responsible for seedlings growth heterosis of rubber tree. The aforementioned results indicate that the hybrids generated from homozygous and heterozygous crossings may show the heterosis by regulating similar GO terms and KEGG pathways. Of course, this speculation need be further testified.

Analyzing gene action models between the two hybrids and their parents, we propose that overdominance (OD or UD) may play important roles on growth heterosis, whereas dominance on hybrid weakness in rubber tree seedlings. It is the first time to analyze growth heterosis and weakness on transcriptome level in rubber tree seedlings. These findings bring new insights into understanding growth heterosis of rubber tree seedling.

## Conclusions

We found that the genotypes, transcriptomes and biological pathways (especially, carbohydrate metabolism) were highly divergent between two hybrids, which may be associated with growth heterosis and weakness. Analyzing gene action models between the two hybrids and their parents, we propose that overdominance may play important roles on growth heterosis, whereas dominance on hybrid weakness in rubber tree seedlings.

## Additional files

Additional file 1: Table S1. Primer pairs designed for validating gene expression. (XLSX 25 kb)

Additional file 2: Table S2. Seedling girth and height of five rubber tree varieties. (XLSX 8 kb)

Additional file 3: Table S3. Gene action models in BT 3410-parent triad. (XLSX 478 kb)

Additional file 4: Table S4. Gene action models in WC 11-parent triad. (XLSX 205 kb)

## Abbreviations

AD: Additivity
GO: Gene ontology
HPD: High parent dominance
KO: KEGG ortholog database
KOG/COG: Clusters of orthologous groups of proteins
LPD: Low parent dominance
NR: Natural rubber
Nr: NCBI non-redundant protein sequences
Nt: NCBI non-redundant nucleotide sequences
OD: Overdominance
Pfam: Protein family
UD: Underdominance
